# IL-18 biology in severe asthma

**DOI:** 10.3389/fmed.2024.1486780

**Published:** 2024-11-01

**Authors:** Sarita Thawanaphong, Aswathi Nair, Emily Volfson, Parameswaran Nair, Manali Mukherjee

**Affiliations:** ^1^Department of Medicine, McMAster University, Hamilton, ON, Canada; ^2^Research Institute of St. Joe’s Hamilton, St. Joseph’s Healthcare Hamilton, Hamilton, ON, Canada; ^3^Division of Pulmonary and Critical Care Medicine, Department of Medicine, Faculty of Medicine, Chulalongkorn University and King Chulalongkorn Memorial Hospital, Thai Red Cross Society, Bangkok, Thailand

**Keywords:** IL-18, autoimmunity, asthma, inflammasome, eosinophilia

## Abstract

The role of interleukin-18 (IL-18) and inflammasomes in chronic inflammatory airway diseases, such as asthma and chronic obstructive pulmonary disease (COPD), has garnered significant attention in recent years. This review aims to provide an overview of the current understanding of IL-18 biology, the associated signaling pathways, and the involvement of inflammasome complexes in airway diseases. We explore the multifaceted role of IL-18 in asthma pathophysiology, including its interactions with other cytokines and contributions to both T2 and non-T2 inflammation. Importantly, emerging evidence highlights IL-18 as a critical player in severe asthma, contributing to chronic airway inflammation, airway hyperresponsiveness (AHR), and mucus impaction. Furthermore, we discuss the emerging evidence of IL-18’s involvement in autoimmunity and highlight potential therapeutic targets within the IL-18 and inflammasome pathways in severe asthma patients with evidence of infections and airway autoimmune responses. By synthesizing recent advancements and ongoing research, this review underscores the importance of IL-18 as a potential novel therapeutic target in the treatment of severe asthma and other related conditions.

## Introduction

Asthma is a common respiratory disease that currently affects 338 million individuals worldwide. The disease’s underlying mechanism of airway inflammation and airway hyperresponsiveness, causing airway wall edema, mucus plugging, airway smooth muscle contraction, and/or airway remodeling, leads to clinical symptoms of chronic, episodic cough, phlegm production, chest tightness, wheezing, and shortness of breath (1). Asthma phenotypes are used to characterize patients based on their clinical features, including symptom severity, a history of exacerbation, and lung function in combination with airway inflammation and genetic profiles (2). Majority of asthma patients with mild to moderate disease are controlled by appropriate treatment with high dose Inhaled corticosteroids/Long-acting beta-2 agonist (ICS/LABA) and comorbidities management. Approximately 5–10% suffer from severe uncontrolled asthma and experience symptoms, persistent airway inflammation, and frequent exacerbations despite high-dose ICS treatment (3). Systemic corticosteroids, which have broad anti-inflammatory effects, remain the mainstay of treatment to manage more severe symptoms but are well known for their adverse effects (4).

Airway inflammation and hyperresponsiveness in asthma occur due to both exogenous and endogenous triggers. The key cytokines involved in the Type 2 inflammatory pathways are IL-5 and IL-13 (5). As a steroid-sparing strategy, anti-T2 monoclonal antibody (mAb) therapies were introduced in the past decade, that have shown modest reduction of asthma exacerbations by 50–60% (6). An optimal, personalized treatment is, therefore needed based on the underlying phenotype and associated endotypes (7). Although blood eosinophil counts are widely used in clinical practice, they may not be sufficient to justify treatment decisions. It is crucial to consider the compartmentalization of airway inflammation in asthma (8). The role of airway eosinophils is further corroborated by a recent cohort study showing sputum eosinophil peroxidase (EPX) is superior to blood eosinophils in understanding patients who remain uncontrolled on anti-IL-5 targeted therapy (9). Again, the modest reduction in exacerbations and persistence of symptoms that remain uncontrolled by anti-IL-5 mAbs (10–12) despite depletion of airway eosinophils suggest alternative/residual airway inflammation (13). The measurement of sputum cytokine in this group of patients revealed raised IL-13, IL-18, and/or IL-1β, indicating alternative pathways beyond IL-5 (14). Furthermore, lack of improvement in asthma symptoms on benralizumab, an eosinophil depleting mAb was associated with raised/residual levels of IL-18 in sputum (13). In severe allergic asthma patients, who typically respond to omalizumab (anti-IgE therapy), high baseline serum free IL-18 levels may predict reduced omalizumab efficacy. A 2-year study showed that significantly more patients showed high baseline serum free IL-18 levels among the patients who developed exacerbation in the second year (incomplete responders) than the complete responder group (15). In non-T2 inflammation, IL-18 works in combination with IL-12, stimulating T helper 1 (Th1) cell development, Natural killer (NK) cells, and Natural killer T (NKT) cells (16). Since IL-18 is primarily produced as an inactive precursor, there is a process to proteolyze pro-IL-18 to an active form. This process is involved in the inflammasome complexes cascade (discussed in detail), which plays an important role in our innate immune systems (17).

This review aims to consolidate current knowledge on IL-18 biology and signaling pathways, particularly the involvement of inflammasome complexes in airway disease to provide active IL-18. We also discuss the role of IL-18 in asthma pathophysiology, autoimmunity, interaction with other cytokines, and its clinical perspective, including the potential therapeutic targets.

## IL-18 biology

IL-18 was initially identified as an interferon-γ (IFN-γ) inducing factor (IGIF). The IGIF is mainly produced as a 193 amino acids precursor and is cleaved to a mature protein of 157 amino acids (18). IL-18 is classified to be a part of the IL-1 cytokine family due to its shared common beta-pleated sheet structure and amino acid sequence (18–20). Similar to the other cytokines in the IL-1 family, IL-18 exerts active functions by binding to the corresponding receptors on the surface of target cells. The IL-18 receptor belongs to IL-1R family members and The Toll/IL-1 receptor (TIR) domain receptors superfamily (21). The extracellular part is conserved to recognize the cytokine while the intracellular part containing a TIR domain orchestrates the downstream signal transduction. The architectural structure of the IL-18 receptor has a second domain that is unique and contributes to the distinct inter-receptor interaction and binding affinity, not present in the other IL-1Rs (22).

The production of IL-18 occurs in a wide array of cell types, including both hematopoietic and non-hematopoietic cells. The IL-18 precursor is present in several cell types, including circulating monocytes, resident macrophages, dendritic cells, endothelial cells, keratinocytes, osteoblasts, intestinal epithelial cells, and mesenchymal cells (23). The IL-18 mRNA or protein is also found in airway epithelial cells (24, 25). Initially synthesized as an inactive precursor, pro-IL-18 undergoes proteolytic cleavage by the intracellular cysteine protease, caspase (Casp)-1, to yield its biologically active form, IL-18, secreted by the cell. This activation process is intricately linked to the canonical inflammasome pathways, particularly Nucleotide-binding oligomerization domain leucine-rich repeat and pyrin domain-containing protein (NLRP) 3, discussed in detail in subsequent sections (17). Additionally, alternative pathways beyond inflammasome are also identified, such as Fas-mediated signaling via Casp-8 in macrophages and dendritic cells (26).

The IL-18 receptor (IL-18R) is expressed in T cells and NK cells. Non-immune cells like neurons and epithelial cells also express IL-18R that may play a role in their cellular differentiation and survival. The receptor comprises of two subunits, IL-18Rα and IL-18Rβ, forming a high-affinity binding heterodimer upon IL-18 stimulation (23). This complex triggers downstream signaling involving myeloid differentiation primary response 88 (MyD88) that activates nuclear factor-κB (NF-κB) and mitogen-activated protein kinase (MAPK) through association with the signal adaptor IL-1R-associated kinase (IRAK) 1–4 and tumor necrosis factor (TNF) receptor-associated factor (TRAF) 6 (27, 28). Inhibitors of IL-18 signaling include IL-18-binding protein (IL-18BP) and IL-37. IL-18BP can bind soluble mature IL-18 with a higher affinity and prevent IL-18 binding to IL-18R. Free IL-37 binds to IL-18α with a low affinity and then induces the recruitment of IL-1R8 to form a high-affinity complex. This complex does not recruit MyD88, thus inhibiting IL-18 signaling and inducing an anti-inflammatory effect via signal transducer and activator of transcription (STAT) 3 (23, 29, 30).

## IL-18 and immunity

### Innate immune response

Innate immunity serves as our first line of defense against a wide array of pathogens, regardless of prior exposure. This system includes physical barriers formed by epithelial cells, phagocytic cells such as neutrophils, macrophages, dendritic cells, and NK cells that target and kill virus-infected and tumor cells, the complement system which enhances pathogen clearance, and the cells that release cytokines that regulate immune responses and inflammation (31).

IL-18 collaborates with IL-12 to stimulate NK cells to produce IL-8, IFN-γ and TNF-α, enhancing their activities against infection and cancer whilst triggering an innate immune response (32). The significance of IL-18 in establishing NK cell activity is evident in IL-18 deficient mice, where susceptibility to infection increases due to impaired NK cell function (33). Additionally, the combined stimulation of IL-18, IL-12, and IL-15 in mice splenic NK cells is associated with the generation of memory-like NK cells, boosting their IFN-γ production when exposed to subsequent repetitive stimuli. This highlights the multifaceted roles of IL-18 in innate immunity (34). Similarly, IL-18, when acting in conjunction with IL-12, activates macrophages, enabling them to produce the crucial cytokine IFN-γ (35). This collaborative action extends to various cell types, including non-polarized T cells, Th1 cells, dendritic cells, and B cells, which can produce IFN-γ in response to the synergistic influence of IL-18 and IL-12 (16).

Airways epithelial cells are the first barrier against inhaled allergens and pathogens. Several extraneous agents, including, fungal agents such as *Alternaria extract* can cause epithelial damage, cell necrosis, and rapid release of IL-18 (36). This was through autophagy-dependent and Casp-1 and Casp-8-independent pathways (37). *Alternaria* sensitization is associated with an increased risk of asthma in children (38). A combination of IL-3 and IL-18 can stimulate mast cells and basophils to produce histamine, IL-4, and IL-13 as an innate allergic response process (39). These interactions underscore the paradigm shift from a response to an adaptive Th2 response in asthma orchestrated by IL-18.

### Adaptive immune response

The adaptive immune response, also known as acquired immunity, is a specific and delayed response that provides long-lasting protection. The key players include B cells and T cells. The latter lymphocytes can be further divided into two main types: Helper T cells (CD4+) and Cytotoxic T cells (CD8+) (31).

In the adaptive immune system, IL-18 plays a pivotal role in the activation and differentiation of T cells. The combination of IL-18 and IL-12 allows a synergized IFN-γ production from T cells and B cells (19). One of the mechanisms to explain this synergistic effect was demonstrated in Th1 cells, where an increase in IL-18R mRNA expression after IL-12 stimulation was observed (40) along with the reciprocal induction/expression of IL-12R by IL-18 (16).

IL-18 further contributes to the immune response by upregulating the cytotoxic activities of NK and CD8+ T cells. These cells, when activated by IL-18, exhibit enhanced capabilities to eliminate target cells through the release of cytotoxic molecules such as perforin or by inducing apoptosis in Fas-expressing target cells (41, 42). Additionally, IL-18 is implicated in the induction of allergic inflammation, by triggering IgE production from B-cells in a CD4^+^ T cell-dependent process via IL-4/STAT6 signaling (43). The NKT cells that express high levels of IL-18Rα, when stimulated with IL-18 in combination with IL-2, can generate a Th2 response with IL-4, IL13 production and induction of CD40 ligand expression (44). Intriguingly, IL-18 has the potential to induce plasticity of Th1 to Th2 cells via upregulation of Th2 transcription factor GATA-binding protein 3. After repetitive stimulation with IL-18 and IL-2, Th1 cells differentiated from IL-13+ IFN-γ+ to cells producing primarily IL-13 (45).

**Figure 1:**
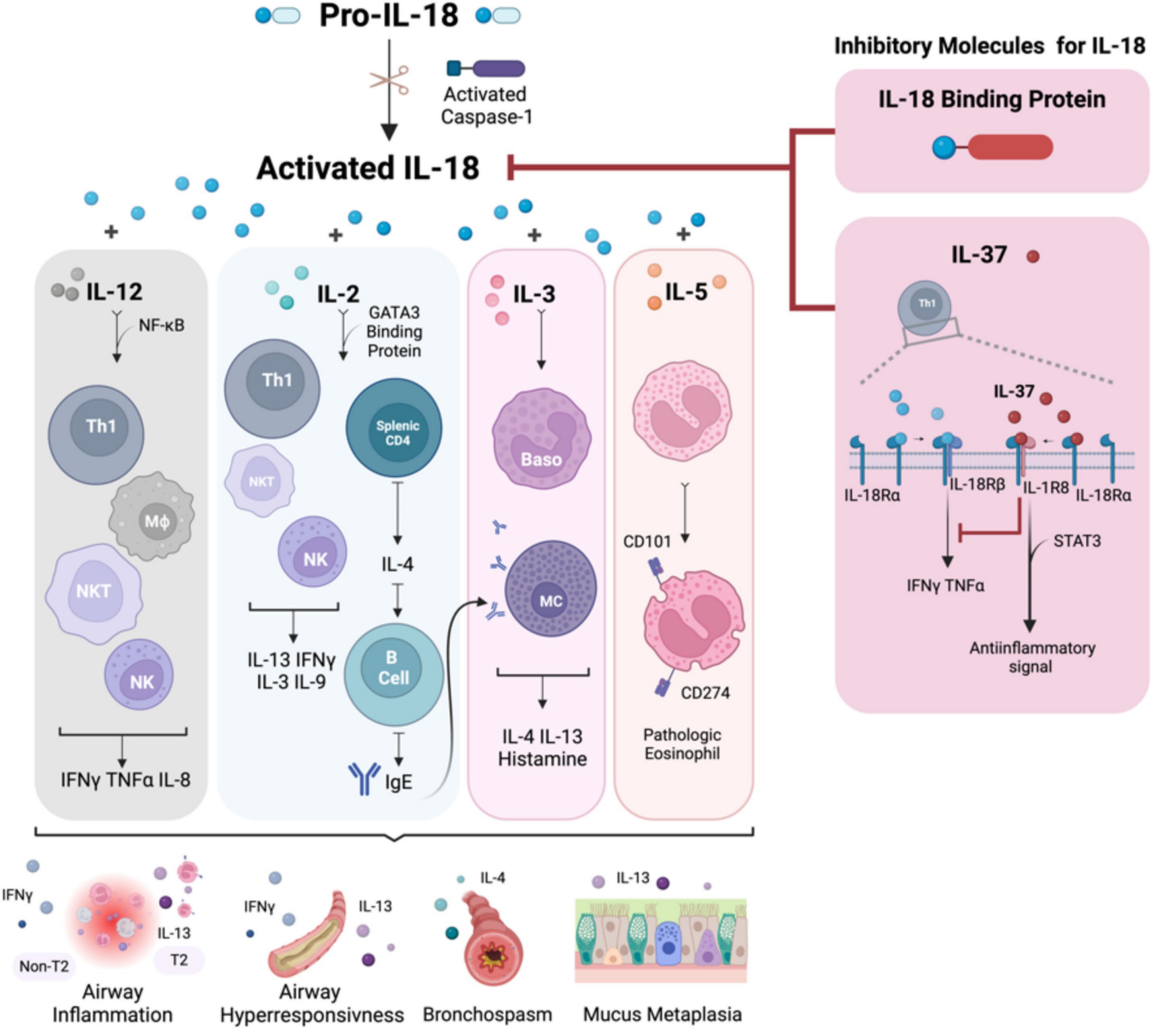
The summary of IL-18 biology and the immune response in association with asthma pathophysiology. IL-18 is activated by caspase-1 and is secreted by both hematopoietic and non-hematopoietic cells. Once activated, IL-18 binds to IL-18R, which is expressed on the surface of T cells, NK cells, neurons, and epithelial cells, triggering downstream inflammatory events. In the Th1 pathway, IL-18, in synergy with IL-12, stimulates Th1 cell development and enhances IFNγ expression. Conversely, in the Th2 pathway, the interaction between IL-18 and IL-2 promotes a Th2 response, which leads to the production of IL-4 and IL-13. This Th2-mediated response increases airway inflammation, hyperresponsiveness, and mucus metaplasia due to IgE production from B cells and heightened IL-13 levels from differentiated cells. Additionally, IL-18 stimulates mast cells and basophils, leading to the release of histamine, IL-4, and IL-13, further promoting a Th2 asthmatic response. IL-18 also induces eosinophils to express CD101 and CD274, transforming IL-5-responsive naive eosinophils into pathogenic eosinophils, contributing to mucus hypersecretion and airway obstruction. IL-18 signaling is regulated by the IL-18 binding protein, which neutralizes IL-18 activity, and inhibitory effects of IL-37, which binds to IL-18α, recruits IL-1R8, and forms a high-affinity complex. This complex inhibits downstream signaling from IL-18 and induces an anti-inflammatory signal via STAT3. Baso, basophil; CD, cluster of differentiation; IFN, interferon; IgE, immunoglobulin E; IL, interleukin; IL-18BP, IL-18 binding protein; MΦ, macrophage; MC, mast cell; NF-κB, nuclear factor kappa-light-chain-enhancer of activated B cells; NK, natural killer; NKT, natural killer T-cell; STAT3, signal transducer and activator of transcription 3; Th, T helper cell; TNF, tumor necrosis factor. Created with Biorender.com.

The biology of IL-18 and its role in the immune response and asthma pathophysiology are summarized in Figure 1. Given the primary signaling pathway of IL-18 expression is via inflammasome activation, the review will next provide an overview of inflammasome biology, and the different clinically relevant triggers that activate this pathway leading to IL18 abundance in tissue.

## Inflammasome

Inflammasomes are intricate cytosolic complexes that are essential components of the innate immune system. They primarily consist of three components: First, a sensory protein or the pattern recognition receptors (PRRs), including the Nucleotide oligomerization domain (NOD)-like receptors (NLRs); second, an adaptor protein called apoptosis-associated speck-like protein containing a Caspase Activation and Recruitment Domain (CARD) (ASC), and third, an enzymatic effector such as Casp1. The NLR family is characterized by the presence of a central nucleotide-binding and oligomerization (NACHT) domain, which is commonly flanked by C-terminal leucine-rich repeats (LRRs) for ligand sensing and N-terminal effector domain for mediating signal transduction. The NLRs can be further categorized into subfamilies based on the effector domains, including the NLRP with pyrin domain (PYD) and NLRC with CARD domain (46, 47). Several inflammasome moieties have been identified thus far: NLRP1, NLRP3, NLRP6/7/12, NLR family CARD domain-containing protein (NLRC) 4, retinoic acid-inducible gene I (RIG-I), absent in melanoma 2 (AIM-2) and interferon gamma-inducible protein 16 (IFI 16). The specific component and activation mechanisms vary depending on the nature of the individual protein.

**Figure 2:**
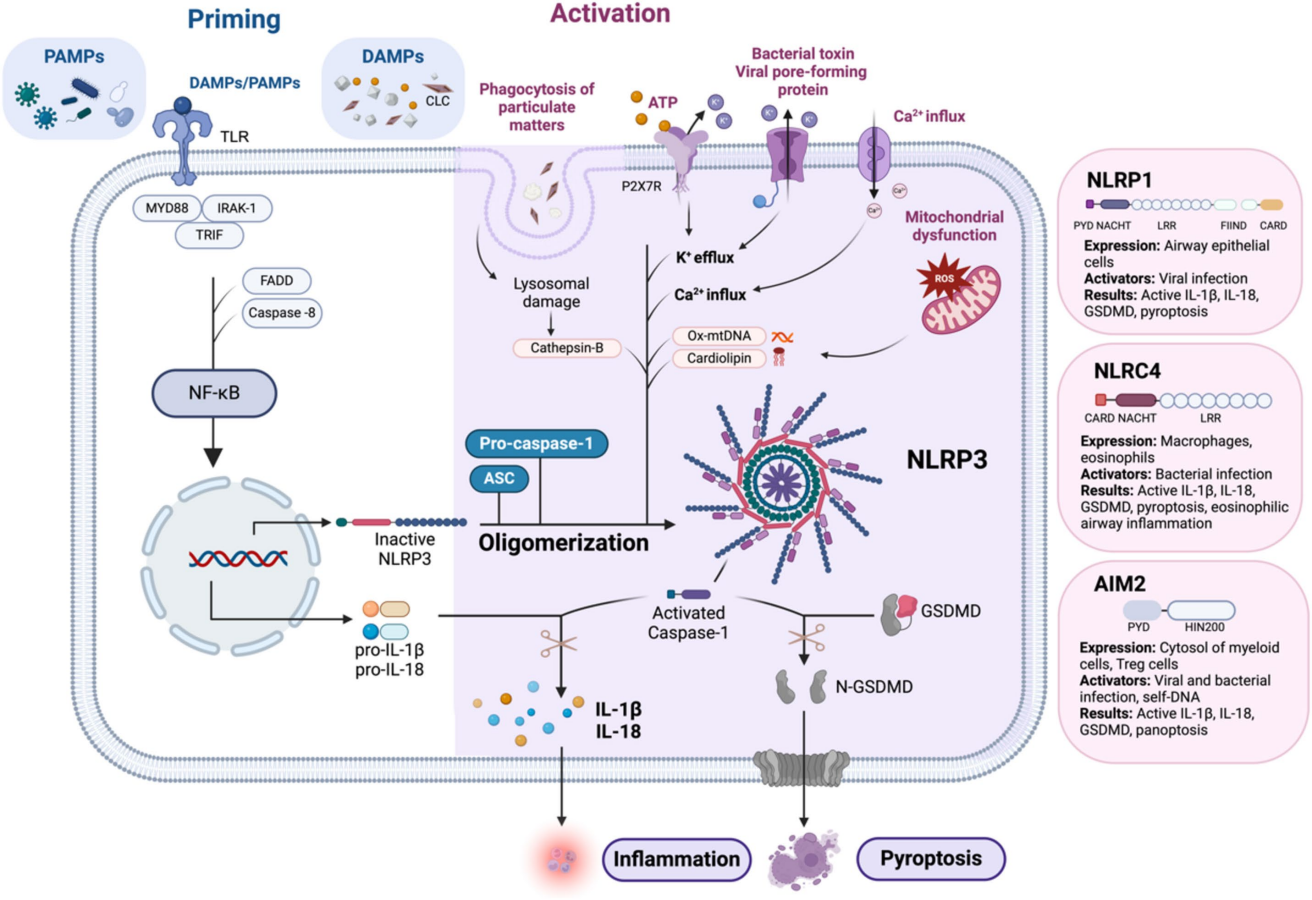
Mechanism of NLRP3 inflammasome activation: the canonical pathway requires two signals: priming and activation. Priming is initiated by the detection of PAMPs and DAMPs, which activate NF-κB by facilitating its translocation into the nucleus via the myddosome complex. This upregulates pro-IL-1β, pro IL-18, and NLRP3 to form the completed NLRP3 inflammasome complex with ASC and pro-Casp-1. Activation of the NLRP3 inflammasome can be triggered by several mechanisms. Phagocytosis of DAMPs can lead to lysosomal damage and the release of cathepsin B. The binding of extracellular ATP to P2X7R, an ATP-gated ion channel, triggers K+ efflux. Additionally, bacterial toxins and viral pore-forming proteins can create cell membrane pores, also leading to K+ efflux. Other mechanisms include Ca2+ influx and mitochondrial dysfunction, which result in the formation of ROS and the secretion of Ox-mtDNA and cardiolipin. Finally, the activation of caspase-1 leads to the conversion of pro-IL-1β and pro-IL-18 into their active forms. Caspase-1 also cleaves GSDMD into N-GSDMD, resulting in pyroptosis. Similar to NLRP3, activation of other airway-relevant inflammasomes, such as NLRP1, NLRC4, and AIM2, also leads to the production of active IL-1β, active IL-18, active GSDMD, and pyroptosis. AIM2, absent in melanoma 2; ASC, apoptosis-associated speck-like protein containing a CARD; ATP, adenosine triphosphate; CARD, caspase recruitment domain; CLC, Charcot-Leyden crystals; DAMPs, damage-associated molecular patterns; DNA, deoxyribonucleic acid; FADD, Fas-associated protein with death domain; FIIND, function-to-find domain; GSDMD, gasdermin D; IL, interleukin; IRAK-1, interleukin-1 receptor-associated kinase 1; LRR, leucine-rich repeat; mtDNA, mitochondrial DNA; MYD88, myeloid differentiation primary response 88; N-GSDMD, N-terminal gasdermin D; NF-κB, nuclear factor kappa-light-chain-enhancer of activated B cells; NLRC4, NOD-like receptor family CARD domain-containing protein 4; NLRP1, NOD-like receptor family pyrin domain-containing protein 1; NLRP3, NOD-like receptor family pyrin domain-containing protein 3; Ox-mtDNA, oxidized mitochondrial DNA; P2X7R, purinergic receptor P2X ligand-gated ion channel 7; PAMPs, pathogen-associated molecular patterns; PYD, pyrin domain; ROS, reactive oxygen species; TLR, toll-like receptor; TRIF, TIR-domain-containing adapter-inducing interferon-β. Created with Biorender.com.

As illustrated in Figure 2, when the sensory protein senses the danger signal, including pathogen-associated molecular patterns (PAMPs), damage-associated molecular patterns (DAMPS), and homeostasis-altering molecular processes (HAMPs) (48), the signaling pathway is activated. ASC is recruited and interconnects with pro-Casp1 through the CARD domain, leading to oligomerization. This process triggers autoproteolysis, giving rise to active Casp1. Active Casp1 plays a central role in cleaving pro-IL-1β and pro-IL-18 into their mature forms, facilitating the release of IL-1β, IL-18, high mobility group box 1 (HMGB1) (a known DAMP), and other proteins from the cell. Casp1 also initiates a specific form of highly inflammatory programmed cell death known as pyroptosis, characterized by rapid plasma membrane rupture, leading to the release of intracellular contents. Unlike apoptosis, which is immunologically silent, pyroptosis serves as a defense mechanism against intracellular pathogens. The term “pyroptosis” derives from the Greek words “pyro,” meaning fire or fever, and “ptosis,” meaning falling, emphasizing both its inflammatory nature and the collapse of cellular integrity (49).

The execution of pyroptosis is mediated by the gasdermin family of proteins, particularly gasdermin D (GSDMD). Upon activation by Casp-1, GSDMD undergoes proteolytic cleavage, liberating its N-terminal domain (50). The N-terminal fragment of GSDMD inserts into the plasma membrane, forming large pores known as “pyroptotic pores.” These pores compromise membrane integrity, leading to osmotic imbalance, cellular swelling, and, ultimately, membrane rupture. Pyroptosis serves as a double-edged sword in host defense, eliminating infected cells to limit pathogen replication while also triggering an inflammatory cascade (51).

Innately, inflammasomes are known for protecting against invading pathogens and initiating adaptive immune responses. However, their dysregulation is implicated in several metabolic disorders (52), autoinflammatory diseases (53, 54), neurodegenerative diseases (55, 56) and of recent, the cytokine storm reported in COVID-19 (57). In the past decade, there has been a growing focus on the role of inflammasomes in chronic airway diseases, particularly asthma and COPD. Mutations of the genes in inflammasome pathway have been associated with eosinophilia in patients with asthma (54). Expression of the NLRP3 inflammasome has been associated with acute exacerbations of COPD (58) and neutrophilic airway inflammation, worsening lung function, and poor asthma control (59). Rhinovirus infection activates RIG-I inflammasome in asthma patients and leads to prolonged viral clearance and unresolved inflammation, demonstrated via both *in-vitro* and *in-vivo investigations* (60).

### NLRP3 inflammasome

The NLPR3 inflammasome consists of the NLRP3 receptor, the adaptor protein ASC, also known as PYCARD, and Casp-1 as an effector protein. The NLRP3 receptor is a tripartite protein that contains an amino-terminal PYD, a nucleotide-binding NACHT, and a carboxy-terminal LRR domain (61). This inflammasome complex is highly expressed in myeloid cells, including monocytes, neutrophils, macrophages, and dendritic cells, associated with immune responses to various types of infection, including virus [Influenza A (62), SARS-CoV-2 (63)], Bacteria (*Listeria monocytogenes*) (64), and fungus [*Candida albicans* (65), *Aspergillus fumigatus* (66)]. Several PAMPs [viral RNA, muramyl dipeptide from bacteria, bacterial RNA and double-stranded RNA, and Galactosaminoglycan from *Aspergillus fumigatus* (67)] and DAMPs [adenosine triphosphate (ATP), uric acid crystals and amyloid-b, silica, asbestos, and alum] are identified as activating agents. In context to the current review topic, Charcot-Leyden Crystals (CLCs), that are essentially Galectin-10 sequestered protein crystals resulting from intense eosinophilic airway inflammation, act as DAMPs and trigger inflammasome NLRP3 in macrophages to release IL-1beta (the study did not assess IL-18) (68). Additionally, HAMPs such as potassium (K+) efflux from bacterial toxin, viral pore-forming protein, P2X7R activation by ATP, GSDMD pore formation, calcium (Ca^2+^) flux, and mitochondrial or lysosomal dysfunction activates NLRP3 (48).

The activation of the canonical NLRP3 inflammasome pathway requires two signals: priming and activation. The priming or transcriptional signal is induced through the NLRs which recognize PAMPs/DAMPs, resulting in the involvement of myddosome complex, which consists of Myeloid differentiation primary response 88 (MyD88), Interleukin-1 receptor-associated kinase 1 (IRAK-1), TIR-domain-containing adaptor-inducing interferon-β (TRIF), Fas-associated protein with death domain (FADD), and Caspase-8. This myddosome complex activates the NF-kB by translocating into the nucleus and further upregulation of NLRP3, pro-IL-1β, and pro-IL-18. Then, the second signal will activate the NLRP3 and assemble it with ASC and pro-Casp-1 to be the NLRP3 inflammasome complex. Many DAMP molecules such as CLCs (68), monosodium urate, silica, asbestos, and amyloid-β, when they are phagocytosed, their physical characteristics can cause lysosomal disruption, releasing their components, including Cathepsin B into the cytoplasm and activate the inflammasome. The PAMPs/DAMPs can trigger the NLRP3 inflammasome complex through reactive oxygen species generation and mitochondrial dysfunction. In addition, there is a noncanonical pathway that responds to intracellular lipopolysaccharides (LPS) of Gram-negative bacteria identified, which is dependent on Casp-4 and 5 (69–71).

### NLRP1 inflammasome

NLRP1 was the first described inflammasome-nucleating protein, also called NACHT, LRR, and PYD domains-containing protein 1 (NALP1), that was involved in the caspase activating complex (72). Similar to other NLRs, the NLRP1 consists of the N-terminal PYD, NACHT domain, and LRRs. However, it has a distinguishing structure where these domains are followed by a function-to-find domain (FIIND) and the C-terminal CARD. The FIIND undergoes autoproteolytic cleavage that generates two fragments: N-terminal region and a C-terminal UNC5, PIDD, and ankyrins (UPA)-CARD domain that remain in an inactive state. The activation process called the functional degradation process, occurs when the N-terminal fragment is degraded by the proteasome to release the UPA-CARD fragment that forms an active inflammasome complex. The result of the activation process leads to inflammation similar to the NLRP3 inflammasome with the involvement of active IL-1β, active IL-18, active GSDMD, and pyroptosis. Expression of NLRP1 has been demonstrated mostly in non-myeloid cells, including human airway epithelial cells (73, 74), human keratinocytes of the skin, and in the lining of gastrointestinal tract (75). The first identified trigger factor for NLRP1 was ribotoxic stress from UV radiation, followed by viral proteases and viral dsRNA (48, 76).

### NLRC4 inflammasome

The NLRC4 is the first inflammasome sensor identified to activate both Casp-1 and cell death. The structure comprises of NLRC4, ASC, and pro-Casp-1 assembled to form the NLRC4 inflammasome complex. Even though ASC is not deemed necessary, the NLRC4 inflammasome complex without ASC shows inefficient Casp-1 cleavage and diminished IL-1β release. The expression of NLRC4 inflammasome is evident in macrophages, eosinophils, and intestinal epithelial cells. It is known to be triggered by bacterial infection. However, the NLRC4 does not detect bacterial components directly. Activation of NLRC4 inflammasome is associated with eosinophilic airway inflammation due to its expression in human eosinophils. In fact, NLRC4-deficient mice have significantly fewer eosinophils in the bronchoalveolar lavage fluid (BALF) as compared to wild-type mice following induction of allergic airway disease (77).

### AIM2 inflammasome

AIM2 terminology expands to “Absent in melanoma 2,” given it was first discovered as a tumor suppressor factor and later found to be involved in the inflammasome pathway. AIM2 is a member of the IFN-inducible HIN-200 family of proteins with an N-terminal PYD and a C-terminal HIN-200 domain. AIM2 exists as an autoinhibited conformation (PYD and the HIN-200 domains) in myeloid cells, keratinocytes, and T regulatory cells. Since its identification as a DNA sensor, AIM2 was found to mediate inflammasome response to bacterial and viral pathogens, including cytomegalovirus (CMV), human papillomavirus (HPV) and *L. monocytogenes*. The double-stranded DNA must be in a minimal length between 70 and 80 bp for the HIN-200 domain recognition, and the binding occurs in a sequence-independent manner. This binding leads to the structural change, which frees the PYD part to assemble with ASC and pro-Casp-1 to form the AIM2 inflammasome complex (48). Activation of AIM2 can lead to the formation of the AIM2-PANoptosome complex, which is implicated in a hybrid cell death pathway known as PANoptosis, involving the simultaneous activation of pyroptosis, apoptosis, and necroptosis in response to *Francisella novicida* and HSV1 infections, resulting in the release of cytokines and DAMPs (78). AIM2 inflammasome activation is also evident in COPD lungs and cigarette-exposed mice with an increase of cleaved IL-1β (79). Additionally, AIM2 has an inflammasome-independent role, as it can bind to neutrophils extracellular traps (NETs), leading to DNase-resistant nucleoprotein fibers that can serve as an autoantigen in SLE (80). This is relevant to severe asthma patients who show evidence of airway autoimmune responses (81), associated with smoking (82) and recurrent infections (83), with evidence of NETs.

## IL-18 in asthma pathophysiology

Even though IL-18 is not recognized to be a major player in asthma pathobiology, several recent studies have linked IL-18 to diverse immune responses in asthma. As discussed earlier, both clinical and basic science investigations suggest IL-18 to play a significant yet complex role in T2 and non-T2 inflammation, depending on the micro-environmental cues (airways). The studies involving IL-18 with respect to asthma, both in animal models and humans, are summarized in Tables 1, 2.

**Table 1:** Summary of clinical studies about IL-18 and asthma in animal models.

First author et al	Year	Populations	Measurements	Results
Kumano et al. (92)	1999	OVA-sensitized mice OVA-sensitized mice with murine rIL-18 intraperitoneal injection	Eo, IL-5, IFN-γ in BALF Airway hyperresponsiveness (acetylcholine challenge)	IL-18 enhances antigen-induced Eo recruitment into the airways but does not affect AHR.
Sugimoto et al. (44)	2004	Unsensitized mice administered with memory type Th1 or Th2 then intranasal administration of Ag+ IL-18 or Ag alone	Inflammatory cells in BALF Cytokines level (IL-4, IL-5, IL-6, IL-9, IL-13, TNF-α, GM-CSF, RANTES, eo-taxin, MIP-1α, and IFN-γ) form supernatants from cultured cells. AHR (methacholine challenge)	Increased eosinophils in BALF and increased AHR in a memory Th1 cells receiving mice with Ag+ IL-18 administration group compare with in those who receiving Ag without IL-18.
Ishikawa et al. (133)	2006	Naïve mice and CD4+ T-cell depletion mice intranasally administered with IL-2 and IL-18	Inflammatory cells in BALF Histopathology of lungs Airway hyperresponsiveness (methacholine challenge)	IL-2 plus IL-18 induced mucus hypersecretion, airway inflammation (increased Eo and Neu in BALF) and AHR in naïve mice but not in CD4+ T cell depletion mice.
Yamagata et al. (115)	2008	OVA/OVA, IL-18 deficient mice OVA/OVA, wild type mice	Cytokines (Il-5, IL-12, IFN-γ, IL-4, IL-13, TGF-β1) from BALF Plasma levels of OVA-specific IgE AHR (acetyl-β-methacholine chloride challenge) Mucus expression in Lung tissues histopathology for mucus expression, peribronchial fibrosis, airway smooth muscle thickness, and number of inflammatory cells.	OVA/OVA IL-18 deficient mice showed a lower level of IL-4, IL-12, IFN-γ, IL-13, and TGF-β1, but not IL-5 in BALF, lower number of infiltrated cells number in lung tissues, lower AHR, fewer mucus expression, fewer peribronchial fibrosis, and fewer smooth muscle thickness from lung tissues compared with OVA/OVA, wild type mice
Kang et al. (106)	2012	IL-18 Tg mice and Wild type mice and were placed on water or Doxycycline water (to stimulate IL-18 production)	Inflammatory cells and cytokine levels (IFN-γ, IL-13, IL-17A, and IL-18) from BALF and whole-lung single-cell suspensions Histopathology of lungs including mucus index	Expression of IL-18 in the lung induces inflammation that is associated with the accumulation of CD4+, CD8+, CD19+, and NK1.1+ cells. IL-18 induced airway fibrosis and mucus metaplasia are mediated by IL-17A and IL-13-dependent mechanism.
Sawada et al. (99)	2013	IL-18 Tg mice and naïve mice, sensitized OVA and challenged with OVA or saline	Inflammatory cells (Neu, Eo, Lym, CD4+ T cells, and CD8+ T cells from BALF) Cytokines (IFN-γ, IL-1β, IL-5, IL-12p70, IL-13, IL-17A/F, and eotaxin from the lungs and BALF) Serum mouse total IgE and OVA-specific IgE levels from AHR (acetylcholine challenge)	Significant increase of inflammatory cells (CD4+ T cells, CD8+ T cells, Eo, Neu and macrophages) in BALF, increase IFN γ, IL-13, and eotaxin, from lungs, and increase AHR in OVA/OVA IL-18 Tg mice compared with OVA/OVA naïve mice In IL-18 Tg mice, overproduction of IL-18 protein in the lungs increased IL-13 producing CD4+ T cells. Administration of anti-CD4 mAb in OVA/OVA IL-18 Tg mice decreased AHR and the levels of IL-13 and IFN-γ in BALF. Deletion of IL-13 gene in OVA/OVA IL-18 Tg mice can showed a decrease Eo in BALF and AHR.
Wang et al. (134)	2016	OVA sensitized mice with intraperitoneal administration of IL-18 with or without IL-18BP and tryptase with or without PAR-2 antagonist peptide	IL-18, IL-4, and TSLP level, IL-18R and PAR-2 expression on mast cells from peritoneal lavage.	IL-18 and tryptase provoked mast cell accumulation, induced an increased in IL-18R+ mast cells, and an increase IL-4 and TSLP.
Mishra et al. (93)	2022	*A. fumigatus*—challenged wild-type mice, CD2-IL-5 Tg mice, CD10-IL-18 Tg mice, IL-5 deficient mice, and IL-18 deficient mice with intranasal administration of rIL-18 or saline.	Pathogenic CD274+ Eo from BALF Airway resistance	IL-18 induces transformation of CD274-Eo to pathogenic CD274+ Eo. Expression of CD 274 in IL-18 deficient mice are significantly reduced compare to IL-5 deficient mice and wild-type mice *In vivo* neutralization of CD 274 and neutralization of IL-18 reduce airway resistance.
Rackov et al. (28)	2022	Memory like CD4+ t cell from mouse spleens stimulated with IL-12/IL-18 or concanavalin (emulating physiological TCR crossing) Diphenyleneiodonium was used for suppress mROS production	Mitochondrial superoxide production (MitoSOX, MitoROS)	IL-12/IL-18 showed faster and augmented mROS production in memory-like cells. mROS inhibition significantly downregulated IFN-γ and CD44 expression. mROS are required for IL-12/IL-18 driven production of IFN-γ.

Ag, antigen; AHR, airway hyperresponsiveness; BALF, bronchoalveolar lavage fluid; CD, cluster of differentiation; Eo, eosinophils; GM-CSF, granulocyte-macrophage colony-stimulating factor; IFN, interferon; IL, interleukin; Lym, lymphocytes; mAb, monoclonal antibody; MIP, macrophage inflammatory protein; mROS, mitochondrial reactive oxygen species; NK, natural killer; Neu, neutrophils; OVA, ovalbumin; PAR, protease-activated receptor; rIL, recombinant interleukin; TCR, T cell receptor; Tg, transgenic; Th, T helper; TGF, transforming growth factor; TNF, tumor necrosis factor; TSLP, thymic stromal lymphopoietin.

**Table 2:** Summary of clinical studies of IL-18 and asthma.

First author et al.	Year	Populations	Measurements	Results
Mild–moderate asthma				
Tanaka et al. (84)	2001	Patients with acute mild or moderate asthma Patients with stable asthma Healthy subjects	Serum IL-18, soluble IL-2 receptor, eosinophil cationic protein, and IFN-𝛾 levels Peak expiratory flow	IL-18 levels were higher in patients with acute asthma IL-18 levels were higher during acute asthma exacerbation than on remission days. IL-18 level had a tendency to inversely correlate with peak expiratory flow.
Imaoka et al. (135)	2011	Patients with allergic asthma Patients with allergic non-asthma Healthy subjects	Serum levels of IL-18, IL-13, IL-4, IL-10, IL-12, and IFN-𝛾 IL-18 protein and IL-18Rα from airway biopsy from allergic asthmatic patient	IL-18 levels were higher in allergic asthma group compared with the others. IL-18 protein was strongly expressed in airway epithelium cells and smooth muscle cells, while IL-18Rα was expressed only on airway epithelium.
Zhang et al. (118)	2018	Patients with asthma Healthy subjects	IL-18, IL-18BP, and IL-18R expression in monocytes, neutrophils, and B-cells.	Increased IL-18 and IL-18BP in asthmatic patients The ratio of plasma level of IL-18 to IL-18BP in asthma patients was 1:12.8. The expression of IL-18BP over IL-18 were 13-fold more in monocytes, 17.5-fold more in neutrophils and 4.1-fold more in B cells from asthmatic blood. Higher IL-18R+ monocytes, neutrophils and B cells are located in asthmatic blood.
Imaoka et al. (85)	2013	Patients with allergic asthma Patients with allergic non-asthma Healthy subjects	Level of soluble IL-18Rα complex and IgE in serum	IL-18Rα complex were higher in allergic asthma group compared with the others. IL-18Rα complex were positively correlated with the serum IgE in overall subject.
Poznanski et al. (32)	2017	*Ex vivo* expanded NK cells from healthy donors – stimulated with IL-18+ IL-12	IL-8 gene expression IL-8 level in cell supernatants	Combined stimulation of IL-18 and IL-12 synergistically upregulates NK cell IL-8 gene expression and increased IL-8 level in supernatants, which was regulated by TNF-α
Murai et al. (37)	2015	Cultured normal human bronchial epithelial cells with ALT-E exposure and in the presence of different inhibitors of autophagy or caspases	Level of IL-18 in cell supernatants The number of autophagosome	ALT-E induced airway epithelial cells to release IL-18 via an autophagy dependent, caspase 1 and 8 independent pathway.
Kubysheva et al. (102)	2020	Patients with asthma, COPD, and ACO Healthy subjects	Levels of IL-17, IL-18, and TNF-α in serum	Higher level of IL-17, IL-18, and TNF-α in all patients compared to healthy subjects In ACO group, the increase in IL-18 levels was associated with the decreased in FEV1.
Murai et al. (36)	2012	Cultured normal human bronchial epithelial cells with ALT-E exposure	Level of IL-18, IL-4, IL-9, IL-13, IL-25, IL-33, or TSLP in cell supernatants	ALT-E can cause epithelial damage, cell necrosis, and rapid release of IL-18
Wu et al. (136)	2024	Patient with asthma Healthy subjects	Levels of N-GSDMD, IL-1β, IL-18, IL-17A, and IL-10 in serum	N-GSDMD, IL-18, and IL-1β were significantly increased in asthma group.
Rodríguez-Alcázar et al. (68)	2019	*Ex vivo* human macrophages from healthy donors	Level of mature IL-1β in cell-free supernatants	CLCs can be phagocytosed by macrophages, leading to the NLRP3 inflammasome activation and release of IL-1β.
Severe asthma				
Oda et al. (86)	2014	Patient with fatal asthma Patient with well-controlled mild asthma Non-asthma patient	IL-18, IL-18R, and Inflammatory cells from lung autopsy and biopsy	Increased IL-18, IL-18R, eosinophils, lymphocytes, CD8+ T cells in the lung from fatal asthma group.
Wang et al. (134)	2016	Atopic asthma patients with acute exacerbation Healthy control	IL-18 and tryptase in serum	Increased IL-18 and tryptase level in moderate and severe asthma patients compared with healthy subjects with significant correlation (*r* = 0.908, *p* < 0.05).
Theofani et al. (21)	2022	*Ex-vivo* CD14+ monocytes from patients with severe asthma and healthy subjects	NLRP3 expression through ASC specks, caspase-1 activation IL-1β and IL-18 levels cell culture supernatants	Higher expression of NLRP3 and higher levels of IL-1β and IL-18 from severe asthma group in non-stimulated state. Significant increase of IL-1β and IL-18 after NLRP3 activation.
Mukherjee et al. (109)	2018	Patients with severe asthma—autoimmune endotype	Cytokines and inflammatory mediators in sputum	Significant increase of IL-18, IL-5, IL-13, eotaxin-2, macrophage-derived chemokine, IL-16, BCA-1, and BAFF in sputum of patients with an autoantibody signature.
Morimoto et al. (15)	2021	Patient with severe allergic asthma, completing 2-year omalizumab treatment	Serum free IL-18 levels before treatment	More incomplete responders, who experienced exacerbations in the second year, had high baseline serum free IL-18 levels (≥141 pg/mL) compared to complete responders.

ACO, asthma-COPD overlap; ALT-E, Alternaria extract; BAFF, B-cell activating factor; BCA-1, B-cell attracting chemokine 1; CD, cluster of differentiation; CLC, Charcot-Leyden crystals; COPD, chronic obstructive pulmonary disease; FEV1, forced expiratory volume in 1 s; IFN, interferon; Ig, immunoglobulin; IL, interleukin; IL-18BP, IL-18 binding protein; N-GSDMD, N-terminal gasdermin D; NLRP3, nucleotide-binding oligomerization domain-like receptor protein 3; OVA, ovalbumin; TSLP, thymic stromal lymphopoietin; TNF, tumor necrosis factor; rIL, recombinant interleukin; PAR, protease-activated receptor; TCR, T cell receptor; ASC, apoptosis-associated speck-like protein containing a CARD.

### Airway inflammation

Significantly higher levels of IL-18 were reported in the serum of asthma patients during exacerbations compared to the stable state. These elevated IL-18 levels did not correspond with changes in IFN-γ levels (84). Significantly increased levels of soluble IL-18Rα complex were seen in the serum of atopic asthmatics compared to allergic non-asthmatics and healthy controls. These levels further correlated with increased IgE serum levels, and the authors suggested an antagonistic activity of IL-18Rα levels in a Th2 allergic response (85). The data from lung autopsy obtained from fatal asthma patients had significant expression of IL-18 protein and IL-18R compared to lung tissues from patients with mild asthma and “no asthma” diagnosis. The levels of eosinophils and lymphocytes but not basophils or macrophages were increased in fatal asthma. Increased numbers of activated CD8^+^ T cells than CD4^+^ T cells were seen in this population (86). In a separate severe asthma cohort, increased levels of NLRP3 pathway components were documented in sputum macrophages from the neutrophilic asthma endotype (87). Additionally, elevated mRNA levels of NLRP3 were observed in CD14^+^ monocytes, along with higher levels of IL-1β and IL-18 in cell culture supernatants. These measurements were taken in the non-stimulated state that showed a further significant increase following NLRP3 activation (88).

IL-5 is a well-established differentiation, growth and survival factor for eosinophils, and earlier reports indicate that IL-5 and eotaxin(s) regulate baseline resident eosinophils. However, IL-18, in combination with IL-5, contribute to its pathogenic characteristics (89). A subset of eosinophils expressing CD101 surface marker was identified as lung-specific inflammatory eosinophils (iEOS) in asthmatic mouse models (90). Again, a subset of CD101+/CD274+ double positive iEOS was identified from nasal lavage of asthma patients. The same study also demonstrates that only IL-18 (not IL-13, IL-15, IL-21, and IL-33) can promote the differentiation and transformation of IL-5 responsive naive eosinophils to pathogenic eosinophils (91). In a murine model, intraperitoneal IL-18 injection increased eosinophil recruitment into the airways (92), and intranasal rIL-18 administration also transformed CD274-eosinophils to CD274+ pathogenic eosinophils that were shown to promote mucus hypersecretion and airway obstruction (93). A recent study demonstrated that IL-18 can transdifferentiate innate lymphoid group 2 cell to atypically express ckit ligand and IL-17, particularly relevant in severe asthma patients with recurrent infections (94). Therefore, IL-18 can orchestrate chronic inflammation in severe asthma beyond the canonical T2 pathways.

### Airway hyperresponsiveness

Airway hyperresponsiveness (AHR) is one of the key diagnostic features of asthma (95). In patients with asthma, even though airway inflammation is not the sole cause of AHR, eosinophilic airway inflammation can contribute to the variable degrees of AHR through the course of the disease (96). IL-18 has differential effect on eosinophilic inflammation and on AHR as demonstrated in animal models. Kumano and co-workers (93) demonstrated an enhancement of airway eosinophilia, but not AHR in sensitized mice by intraperitoneal administration of recombinant IL-18. This was likely mediated by TNF and not IL-5. Further evidence that the effect of IL-18 is through a Th1 pathway was provided by Sugimoto et al. who administered memory-type Th1 and Th2 cells to non-sensitized mice to avoid the background response of host-derived T-cells. The mice that received Th2 cells developed both airway inflammation and AHR after antigen induction, whereas mice that received Th1 cells exhibited airway inflammation but did not develop AHR. However, the co-administration of IL-18 in the Th1 cell-recipient mice induced both airway inflammation and AHR, highlighting the role of IL-18 in driving AHR, within the context of Th1 cell activity (97). It was demonstrated in a latter study that this process is associated with IL-13 and IFNγ production. Not only can exogenous IL-18 induce AHR, but also the endogenous IL-18 induced by lipopolysaccharide (LPS), can cause the same effect (98). The study in ovalbumin-sensitized and challenged transgenic mice show IL-18 to cause significant increases in AHR and airway inflammatory cells, including CD4+ T cells, CD8+ T cells, eosinophils, neutrophils, and macrophages (99).

### Lung function, airway obstruction and mucus impaction

Since asthma is a disease of variable airflow obstruction, patients can have fluctuating lung function over time. However, some patients, usually with long-standing disease, a history of severe exacerbation and/or lack of appropriate treatment, might develop airway remodeling, leading to lung function decline and irreversible airflow obstruction (100, 101). The role of IL-18 or even an association with lung function in asthma is ill-defined. A few studies suggest IL-18 may underlie lower FEV1, but any direct role has not been discerned. For instance, an increase in serum IL-18 levels was documented with a decrease in FEV_1_ in the patients with asthma-COPD-overlap (102). Data from the Severe Asthma Research Program (SARP) cohort using machine learning validated *IL18R1* protein expression in lung tissue and identified downstream NF-κB and activator protein 1 (AP-1) activity. IL-18R1 was negatively correlated to FEV_1_ in both the SARP and Immune Mechanisms of Severe Asthma (IMSA) cohort (103).

IL-18 may indirectly affect airway obstruction by contributing to mechanisms of mucus plugging. Indeed, mucus impaction underscores airway obstruction (104, 105). A 2012 study by Kang and colleagues found that mucus metaplasia, as well as airway fibrosis and vascular remodeling, can be induced by IL-18 via IL-13 and IL-17A cytokines and not IFN-γ. Further, IL-18 transgenic (Tg) mice that lacked IL-17A and/or IL-13 had a significant decrease in airway fibrosis and mucus metaplasia. Alternately, airway fibrosis and mucus metaplasia increased significantly in IL-18 Tg mice that lacked IFN-γ (106).

## Asthma, autoimmunity, and IL-18

Autoimmunity arises when the body’s immune system mistakenly identifies its own cells as foreign, leading to the production of autoantibodies against self-structures. This self-reactivity results in sustained self-immune response and tissue damage (107). The consequence of this phenomenon is demonstrated in a variety of diseases ranging from systemic involvement, such as systemic lupus erythematosus, to organ-specific pathology, such as Crohn’s disease and Hashimoto’s thyroiditis. Even though autoimmunity is theoretically related to Th1/Th17 responses, its possible involvement in the pathogenesis/severity of Th2 diseases such as asthma and chronic rhinosinusitis is being extensively studied (107). Chronic inflammation and subsequent inflammasome signaling may underscore the development and sustenance of airway autoimmune responses described in the airways of patients with complex airways disease (107–109).

In patients with severe asthma with increased airway degranulation evident by the presence of free eosinophil granules, autoantibodies to eosinophil granule proteins such as eosinophil peroxidase (EPX) and anti-nuclear/extranuclear antigens (ANAs) have been reported (81, 109). These sputum autoantibodies were observed in an IL-13-rich micro-environment with increased levels of IL-18 (109). The autoantibodies trigger eosinophil extracellular traps (a similar event to NETs) termed EEtosis (109, 110) which is associated with the release of HMGB1 and crystallization of the periplasmic Galectin-10 to form CLCs (111, 112). The DAMPs activate inflammasomes, leading to the subsequent release of active IL-18 (113), and propagate inflammation and tissue damage beyond the canonical IL-5 and IL-13 pathways. This process leads to self-antigens and activation of self-reactive lymphocytes, resulting in the production of autoantibodies (107), which further triggers and perpetuates EETosis, resulting in a vicious cycle of chronic persistent inflammation in severe asthma (107). Inflammasome signaling and associated IL-18 release may also underlie some of the suboptimal responses documented in prototype severe eosinophilic patients treated with anti-IL-5 (10)/IL-5R biologics (12, 13, 114).

## Potential therapeutic targets of IL-18 and Inflammasome in asthma

Recent research has increasingly focused on the role of IL-18 and inflammasomes in a variety of autoimmune and inflammatory diseases, including chronic lung diseases such as asthma and COPD. The IL-18 and inflammasome cascade play crucial roles in the immune response, with IL-18 being intricately involved in both T2 and non-T2 airway inflammation. This has highlighted IL-18 and related proteins as potential therapeutic targets for severe asthma beyond conventional T2 targets.

Animal models have provided encouraging data, indicating that IL-18 suppression can reduce airway inflammation, AHR, and mucus production (93, 115). Therapeutic strategies aimed at inhibiting IL-18 is either by directly blocking its signaling or target its activation/release by modulating the inflammasome pathway. Various molecules have been studied in diverse inflammatory conditions associated with the IL-18 pathways, offering potential avenues for treatment (tabulated in Table 3). For example, the humanized monoclonal antibody GSK 1070806 has been tested in renal transplant delayed graft function (116), Type 2 diabetes patients (117) and is currently in Phase II developmental pipeline for atopic dermatitis indication (NCT05999799). The search for therapeutic targets has now extended to IL-18R blocking agents and IL-18BP activity-enhancing therapies (118). IL-37, which binds to the IL-18Rα, has also shown potential in asthma treatment. Local administration of IL-37 in asthmatic mouse models reduced eosinophils in the airway and improved AHR (119). Another promising therapeutic, APB-R3, a long-acting recombinant human IL-18BP, has shown effectiveness in reducing liver inflammation and splenomegaly in a model of the macrophage activation syndrome and controlled skin inflammation in atopic dermatitis mice model (120). Tadekinig alfa, a recombinant IL-18 binding protein, has completed Phase II trials in adult-onset Still’s disease, showing early signs of efficacy (121).

**Table 3:** Summary of clinical studies targeting IL-18/inflammasome.

Molecule/drug name & manufacturer	Dose and route	Mechanism of action	Clinical studies on other diseases and outcomes with doses	Clinical studies on respiratory diseases and outcomes
GSK 1070806 (GlaxoSmithKline)	Dose used: 0.25 mg/kg and 5 mg/kg 3 mg/kg 2 mg/kg Route: IV Current clinical dose: Not yet approved	Humanized anti-IL-18 monoclonal IgG_1_ antibody	**Type 2 diabetes**: Phase IIa study showed that GSK10708 did not improve glucose control (117) **Delayed graft function after kidney transplant**: Phase IIa study found that GSK1070806 was unlikely to reduce the risk of DGF (116) **Atopic dermatitis**: Phase Ib study showed a positive treatment effect on clinical score and patient-reported outcomes (From Abstract No 4304; 32nd EADV congress 2023) **Atopic dermatitis:** Ongoing Phase IIb study (NCT05999799).	*No clinical studies on respiratory diseases available.*
APB-R3 (AprilBio Co., Ltd.)	Dose used: 3 mg/kg and 10 mg/kg 1 mg/kg 10 mg/kg Route: IV and IP Current clinical dose: Not yet approved	Long-acting recombinant human IL-18BP	**Macrophage activation syndrome**: Preclinical study showed a reduction in liver inflammation and splenomegaly (120) **Atopic dermatitis**: Preclinical study demonstrated controlled skin inflammation (120) **Primary sclerosing cholangitis**: Preclinical study showed a reduction in periductal fibrosis and transcriptional expressions of pro-fibrotic marker genes (137) **Healthy Subjects**: Completed Phase I study (NCT05715736).	*No clinical studies on respiratory diseases available.*
Tadekinig alfa (AB2 Bio Ltd)	Dose used: 80 mg or 160 mg Route: Subcutaneous injection Current clinical dose: Not yet approved	Human recombinant IL-18BP	**Adult-onset Still’s Disease**: Phase II study indicated a favorable safety profile with preliminary efficacy (121) **CAR T Cell Related Cytokine Release Syndrome and HLH-like Syndrome**: Ongoing early Phase I study (NCT05306080). **NLRC4 Mutation and XIAP Deficiency**: Ongoing Phase III studies (NCT03113760, NCT03512314).	*No clinical studies on respiratory diseases available.*
Selnoflast (RO7486967) (Hoffmann-La Roche, Inflazome Ltd.)	Dose used: 450 mg QD Route: N/A Current clinical dose: Not yet approved	Potent, selective, and reversible NLRP3 inhibitor	**Ulcerative colitis**: Phase Ib study showed a favorable safety profile but no significant changes in plasma IL-18 levels (122) **Parkinson’s disease**: Ongoing Phase Ib study (NCT05924243). **Coronary Heart Disease**: Ongoing Phase Ic study (GC43343).	**Asthma**: Ongoing Phase Ib study (ISRCTN73873157 and EUCT 2023-504304-29-00) **COPD**: Phase Ib study (Completed: EUCTR 2021–000558-25-NL)
MCC 950 (CP-456,773) (AdipoGen, Selleck Chemicals LLC, Sima Aldrich)	Dose used: 40 mg/kg 50 mg/kg 10 mg/kg 20 mg/kg (*in vivo*) and 10 μM (*in vitro*) 1 mg/kg (low dose) and 10 mg/kg (high dose) 50 μg/g Route: Oral and IP Current clinical dose: Not yet approved	NLRP3 inflammasome activation inhibitor	**Chronic Colitis**: Preclinical study demonstrated suppression of proinflammatory cytokines, including IL-1β, IL-18, and IFNγ (138) **Cryopyrin-associated periodic syndrome**: Preclinical study found that MCC 950 failed to inhibit NLRP3-driven inflammatory pathology (139) **Doxorubicin-induced myocardial injury**: Preclinical study demonstrated improved myocardial function, inhibition of inflammation and myocardial fibrosis, and attenuation of cardiomyocyte pyroptosis (140) **Cerebral ischemia–reperfusion injury:** Preclinical study showed that MCC950 effectively reversed NLRP3 inflammasome activation and neuronal pyroptosis (141)	**Asthma**: Preclinical study showed suppression of neutrophilic airway inflammation (59) **Neutrophilic asthma**: Preclinical study demonstrated reduction of airway inflammation and AHR (142) **Allergic rhinitis:** Preclinical study demonstrated enhanced PINK1/Parkin-mediated mitophagy, reduced inflammation, oxidative stress, and apoptosis (143)
Ac-YVAD-cmk (Sigma Aldrich)	Dose used: 12.5 μmol/kg 0.2 mg/mL and 5 mL/100 g 5 μg/g 6.5 mg/kg Route: IP Current clinical dose: Not yet approved	Selective caspase-1 inhibitor	**Sevoflurane-induced cognitive dysfunction**: Preclinical study demonstrated reduction of caspase-1, IL-1β, IL-18 and NLRP3 inflammasome activation, ameliorated learning ability impairment, and reversed the mitophagy flux dysfunction (144) **Sepsis-induced acute kidney injury**: Preclinical study showed decrease expression of Caspas-1, NLRP-1, IL-1β, IL-18, and GSDMD in renal tissues (145)	**Neutrophilic asthma**: Preclinical study showed the reduction of airway inflammation and airway hyperresponsiveness (142) **Acute Respiratory Distress Syndrome**: Preclinical study showed the attenuated NET levels in BALF and neutrophil infiltration in alveoli (146)
Sodium houttuyfonate (Shanghai Qingping Pharmaceutical Co., Ltd., Dalian Meilun Biotechnology Co., Ltd)	Dose used: 50 mg/kg/d (low dose) and 100 mg/kg/d (high dose) 10 mg/kg Route: IP Current clinical dose: Not yet approved	Anti-inflammatory	**Diabetic cardiomyopathy**: Preclinical study showed attenuated cardiac injury caused by hyperglycemia and improve blood lipid (147)	**Asthma**: Preclinical study showed reduction of the expression of NLRP3, ASC, caspase-1, GSDMD, IL-1β, and IL-18 in the lung tissues (125)
Isoquinoline alkaloid protopine (Sigma Aldrich, Beijing Solarbio Science & Technology)	Dose used: 10 μM, 20 μM, 40 μM 10 mg/kg, 20 mg/kg, 40 mg/kg Route: IV and gavage in DMSO and water mixture Current clinical dose: Not yet approved	Anti-inflammatory	**Liver carcinoma**: Preclinical study demonstrated a suppression of tumor growth (148)	**Asthma**: Preclinical study showed a suppression of NLRP3, GSDMD, and caspase-1 activation, decreased levels of ROS and IL-1β and IL-18 (127)
Liraglutide (MedChemExpress, Novo Nordisk)	Dose used: 200 μg/kg/day 1 mg/kg and 2 mg/kg 3 mg Route: IP and subcutaneous injection with saline Current clinical dose: Not yet approved	GLP-1R agonist	**Cognitive impairment in T2DM**: Preclinical study showed that liraglutide can reduce the secretion of IL-1β, IL-18, and TNF-α, and reduces neuroinflammation by influencing astrocyte behavior (149)	**Asthma with obesity**: Preclinical study showed a reduction in the expression of NLRP3, activated caspase-1, and IL-1β in lung tissues, and suppression of AHR (128) **COPD with obesity**: Phase II study showed improvement in symptoms score, FVC and DLCO (150) **Acute lung injury:** Preclinical study showed the liraglutide can reduce the IL-1β and IL-18 levels in BAL fluid, and inhibit the expression of NLRP3 inflammasome (151)
miR-223 agomirs (Shanghai Jima Biotechnology Co., Ltd., RiboBio)	Dose used: 200 μL agomir (1 nm/mouse) 5 nmol miR-223 agomirs in 50ul saline Route: Subcutaneous injection and Intranasal administration Current clinical dose: Not yet approved	microRNA	**Osteoarthritis**: Preclinical study demonstrated that exogenous miR-223 can inhibit NLRP3 inflammasome activation and chondrocyte pyroptosis, showing promising results for the treatment of OA (152) **Acute gouty arthritis**: Preclinical study demonstrated that miR-223-3p can inhibit NLRP3 expression, leading to a reduction in gouty inflammation (153)	**Neutrophilic asthma**: Preclinical study demonstrated attenuated airway inflammation, reduced NLRP3 levels, and decreased IL-1β release (132)

AHR, airway hyperresponsiveness; ASC, apoptosis-associated speck-like protein containing a CARD; BALF, bronchoalveolar lavage fluid; CAR-T, chimeric antigen receptor T cell; COPD, chronic obstructive pulmonary disease; DGF, delayed graft function; DLCO, diffusion capacity of the lungs for carbon monoxide; DMSO, dimethyl sulfoxide; FVC, forced vital capacity; GSDMD, gasdermin D; GLP-1R, glucagon-like peptide-1 receptor; HLH, hemophagocytic lymphohistiocytosis; IL, interleukin; IL-18BP, interleukin-18 binding protein; IP, intraperitoneal; IV, intravenous; mAb, monoclonal antibody; miR, microRNA; NET, neutrophil extracellular trap; NLRC4, nucleotide-binding oligomerization domain-like receptor C4; NLRP3, nucleotide-binding oligomerization domain-like receptor protein 3; OA, osteoarthritis; ROS, reactive oxygen species; T2DM, type 2 diabetes mellitus; TNF, tumor necrosis factor.

Inflammasomes, particularly NLRP3, that cleaves precursor forms of IL-1β and IL-18 into their biologically active forms, have also emerged as therapeutic targets. Numerous agents are under investigation, including Selnoflast, a potent, selective, and reversible NLRP3 blocker, which has completed a Phase Ib study in ulcerative colitis patients (122). Ongoing studies are exploring its potential in diseases such as Parkinson’s (NCT05924243), COPD, and asthma (ISRCTN73873157). Another small-molecule inhibitor, MCC 950 (CP-456,773), a diaryl sulfonylurea-containing compound, that specifically targets NLRP3 inflammasome activation (123) documented significant reductions in NLRP3 and IL-1β production in asthmatic mice (124). Furthermore, sodium houttuyfonate, derived from the Chinese herb *Houttuynia cordata*, has demonstrated efficacy in reducing the expression of NLRP3, ASC, caspase-1, GSDMD, IL-1β, and IL-18 in the lung tissues of asthmatic mice (125). Similarly, the isoquinoline alkaloid protopine, an anti-inflammatory agent (126), has been shown to reduce airway inflammation in asthmatic rats by inhibiting the TLR4/NF-κB signaling pathways, leading to the suppression of NLRP3, gasdermin D, and caspase-1 activation, along with decreased levels of reactive oxygen species (ROS), IL-1β and IL-18 (127).

Another promising therapeutic approach involves the use of glucagon-like peptide-1 receptor (GLP-1R) agonists, initially approved as anti-diabetic and anti-obesity drugs, have been studied for their potential benefits in obesity-related asthma. Obesity is a major risk factor and disease modifier in asthma (125), and studies in obese asthmatic mouse have revealed that AHR can be NLRP3-dependent. GLP-1R agonists have been shown to suppress peri bronchial inflammation and reduce the expression of NLRP3, activated caspase-1, and IL-1β in lung tissues (128). Additionally, miR-223, a microRNA with evolutionary anti-inflammatory effects, particularly in the lungs, has shown potential as a therapeutic option (129). The microRNA, a non-coding RNA, can function to control the expression of target genes at the post transcriptional level. Overexpression of miR-223 is linked to decreased NLRP3 and NF-κB activity in porcine lungs (130) and bronchial epithelial cells (131). Treatment with miR-223 agomirs in neutrophilic asthmatic mouse models attenuates airway inflammation, reduces NLRP3 levels, and decreases IL-1β release, suggesting miR-223 as a potential therapeutic candidate for severe non-T2 asthma (132).

## Conclusion

Ongoing investigations in IL-18 and inflammasomes have revealed their critical roles in the pathophysiology of asthma, particularly in severe forms of the disease. IL-18, traditionally seen as a minor player compared to the key T2 cytokines like IL-5, IL-4, and IL-13, have emerged as a key cytokine involved in both T2 and non-T2 inflammation. Its role in promoting infections, airway inflammation and airway remodeling, as well as its contribution to autoimmune responses within the airways, underscores the complexity of asthma beyond the conventional Th2 paradigm. In this review we have highlighted the multifaceted involvement of IL-18 in asthma, from its interactions with other cytokines to its activation through inflammasome pathways, particularly NLRP3. The evidence linking IL-18 to both airway inflammation, mucus plugging and AHR emphasizes its significance in asthma pathogenesis. Additionally, IL-18’s involvement in autoimmune processes introduces a new dimension to understanding severe asthma, particularly in cases where traditional therapies targeting Th2 inflammation have shown limited effectiveness (107). Furthermore, the current review sheds light on potential therapeutic targets. The identification of novel therapies targeting IL-18 and the associated inflammasome pathways offer promising avenues for treating severe and refractory asthma, addressing the limitations of current biologics. In conclusion, IL-18 is not just an ancillary cytokine in asthma but one of the key components in its pathophysiology, particularly in severe cases. A deeper understanding of its intricate role within the broader network of immune responses can guide the development of more effective treatments, offering new hope for patients with this challenging condition.
