# Corrigendum: IL-18 biology in severe asthma

**DOI:** 10.3389/fmed.2025.1558229

**Published:** 2025-01-21

**Authors:** Sarita Thawanaphong, Aswathi Nair, Emily Volfson, Parameswaran Nair, Manali Mukherjee

**Affiliations:** ^1^Department of Medicine, McMAster University, Hamilton, ON, Canada; ^2^Research Institute of St. Joe's Hamilton, St. Joseph's Healthcare Hamilton, Hamilton, ON, Canada; ^3^Division of Pulmonary and Critical Care Medicine, Department of Medicine, Faculty of Medicine, Chulalongkorn University and King Chulalongkorn Memorial Hospital, Thai Red Cross Society, Bangkok, Thailand

**Keywords:** IL-18, autoimmunity, asthma, inflammasome, eosinophilia

In the published article, there was an error in **Table 3**. In Row 2, Column 1, the manufacturer information for APB-R3 is incorrectly listed as “Sigma-Aldrich.” The corrected Table 3, Row 2, Column 1 appear below.

**APB-R3 (AprilBio Co., Ltd.)**

In Row 2, Column 4, the status of the study in Healthy Subjects was previously noted as “Ongoing Phase I study (NCT05715736).” The corrected **Table 3**, Row 2, Column 4, the fourth bullet appear below.

**Healthy Subjects: Completed Phase I study (NCT05715736)**.

The authors apologize for this error and state that this does not change the scientific conclusions of the article in any way. The original article has been updated.

